# Inhibitory Effect of Polyclonal Antibodies Against HER3 Extracellular Subdomains on Breast Cancer Cell Lines

**DOI:** 10.31557/APJCP.2020.21.2.439

**Published:** 2020

**Authors:** Samaneh Mansouri-Fard, Mojgan Ghaedi, Mohammad-Reza Shokri, Tannaz Bahadori, Jalal Khoshnoodi, Forough Golsaz-Shirazi, Mahmood Jeddi-Tehrani, Mohammad Mehdi Amiri, Fazel Shokri

**Affiliations:** 1 *Department of Immunology, School of Public Health, Tehran University of Medical Sciences, *; 2 *Department of Immunology, School of Medicine, Iran University of Medical Sciences, *; 3 *Monoclonal Antibody Research Center, Avicenna Research Institute, ACECR, Tehran, Iran. *

**Keywords:** Breast cancer, fusion protein, HER3, polyclonal antibody

## Abstract

**Objective:**

Human epidermal growth factor receptor 3 (HER3) is a unique member of the tyrosine kinase receptors with an inactive kinase domain and is the preferable dimerization partner for HER2 which lead to potent tumorigenic signaling.

**Methods:**

In this study, the expression plasmids coding for the human HER3 subdomains were transfected into CHO-K1 cells. Produced proteins were characterized by ELISA and SDS-PAGE. Rabbits were immunized and produced polyclonal antibodies (pAbs) that were characterized by ELISA, Immunoblotting and flowcytometry and their inhibitory effects were assessed by XTT on BT-474 and JIMT-1 breast cancer cell lines.

**Result:**

The recombinant subdomains were highly immunogenic in rabbits. The pAbs reacted with the recombinant subdomains as well as commercial HER3 and the native receptor on tumor cell membranes and could significantly inhibit growth of Trastuzumab sensitive (BT-474) and resistant (JIMT-1) breast cancer cell lines in vitro.

**Conclusion:**

It seems that HER3 extra cellular domains (ECD) induce a strong anti-tumor antibody response and may prove to be potentially useful for immunotherapeutic applications.

## Introduction

Epidermal growth factor receptors (EGFRs) are expressed on the surface of the mammalian cells and play crucial roles in cell division, migration, survival, differentiation and development in the embryonic and adult tissues. Uncontrolled signalling of these receptors accounts for various cancers. This family belongs to subclass 1 of receptor kinase super family and have four members: HER1 (ErbB1 or EGFR), HER2 (ErbB2), HER3 (ErbB3) and HER4 (ErbB4) (Hynes and Lane, 2005; Zahnow, 2006). The ErbB receptors have a general structure in common that includes: an extracellular ligand-binding domain, a transmembrane single helix region, and an intracellular kinase domain (Needham et al., 2016).

Pioneering studies have shown that these receptors are relevant to at least eleven EGF-related peptide growth factors and they have been divided into three groups. The first group binds only to HER1 and includes EGF, TGF-α, and amphiregulin (AR). The second group binds to HER1 and HER4 and includes betacellulin (BTC), heparin-binding EGF (HB-EGF), and epiregulin (EPR). The third group includes Neuregulin-1 (NRG-1) and NRG-2 that bind to both HER3 and HER4 and NRG-3 and NRG-4 that bind only to HER4 (Zahnow, 2006).

Extracellular domains (ECD) in all receptor tyrosine kinases (RTKs) consist of 4 regions or subdomains named DI, DII, DIII and DIV. DI and DIII are leucine-reach and responsible for ligand attachment. DII and DIV with high levels of cysteine residues, are responsible for di-sulfide bond formation and receptor consistency (Olayioye et al., 2000; Cho and Leahy, 2002).

Ligand binding to ECD serves as an activation factor and induces homo- or heterodimer formation and following kinase domain activation, it phosphorylates specific tyrosine residues. These phosphorylated residues recruit some adaptor proteins which can activate downstream signalling (Olayioye et al., 2000). 

Contrary to the other HER receptors, HER3 has an inactive kinase domain. In the human, chromosome 12q13 encodes the HER3 genes which is translated to a 185 kDa glycoprotein (Kraus et al., 1989; SIERKE et al., 1997). This receptor can be overexpressed in some cancers and studies have proven the importance of HER3 in malignancies such as melanoma, breast, lung, prostate, ovarian and colorectal cancers (Amin et al., 2010; Ma et al., 2014; Zhang et al., 2015). 

Due to the inactive kinase domain, homo-dimer formation in HER3 receptor cannot induce an active downstream signal, so it is the best partner for heterodimer complex formation. This receptor has also been shown to preferentially pair with HER1 and HER2 and is overexpressed in cancers along with HER1 and HER2. Furthermore, since HER2 has no known ligand and is always in an open conformation, HER3 is considered as a necessary dimerization partner for HER2 and is the strongest one among all heterodimers (Holbro et al., 2003; Schulze et al., 2005; Jones et al., 2006; Ma et al., 2014; Miller et al., 2014). Dimerization of HER3 with HER2 may lead to HER2 targeted treatment failure and multi-drug resistance in cancers (Schulze et al., 2005; Ma et al., 2014).

Considering HER molecular structures and their importance in tumor pathogenesis, different modalities of targeted therapies have been developed. Several monoclonal antibodies have so far been produced against HER3. These monoclonal antibodies are in different phases of clinical trial but none of them have been able to acquire FDA approval, yet (Jiang et al., 2012; Gaborit et al., 2015). Parallel attempts to develop appropriate immunotherapeutic approaches for induction of active immune responses are noticeable but few studies have been devoted to HER3.

The main objective of this study was to evaluate the antibody response to different subdomains of HER3 extracellular domain (HER3-ECD) and the anti-tumor activity of these antibodies on HER3 expressing tumor cells. To this end, we produced recombinant paired subdomains of DI+II and DIII+IV as well as full HER3-ECD proteins in CHO host cells. Subsequently, rabbits were immunized with recombinant proteins and then purified polyclonal antibodies were used for assessment of their inhibitory effects on HER2/HER3 expressing tumor cell lines (BT-474 and JIM-T1).

## Materials and Methods

*Production of expression constructs*

Extracellular domain and subdomain genes of human HER3-ECD were amplified using specific primers (Table 1) and a vector containing full length of HER3 coding sequence as a template (kindly provided by Professor Bridget S. Wilson) (Steinkamp et al., 2014). For the purpose of detection and purification, the domains were fused to mouse IgG2a region gene. The PCR products were subcloned into pSecTag2/hygro eukaryotic expression vector (OriGene, Maryland, USA) which had already contained the sequence of mouse IgG2a Fc fragment (prepared in our lab) using SmaI and BstBI restriction enzymes. All final constructs were verified by automated sequencing before they were applied for transfection.

*Transfection, expression and purification of the fusion proteins*

We cultured CHO-K1 cells (National Cell Bank of Iran, Pasteur Institute, Iran) were cultured in RPMI-1640 medium (Gibco, California, USA) supplemented with 10% heat inactivated fetal bovine serum (Gibco, California, USA), 1% penicillin (100 IU/ml) and streptomycin (100 μg/mL) (Gibco, California, USA) and used lipofectamine 2000 (Invitrogen, California, USA) in a protocol recommended by the manufacturer to transiently transfect CHO-K1 cells. We seeded 2.5×10^5 ^CHO-K1 cells in every well of 12-well plates. Next day, after washing the cells with pure RPMI or sterile 1X PBS, 300 µl of Opti-MEM medium (Invitrogen) was added to every well. Four µg of each construct were diluted in 100 µl Opti-MEM (Invitrogen) and besides, 3 µl lipofectamine 2000 were added to 100 µl Opti-MEM. Opti-MEM medium containing DNA was added to the mixture of Opti-MEM and lipofectamine 2000. After 5-20 minutes incubation at room temperature, the lipid-DNA mixture was added to each well. Then, the cells were incubated at 37°C in 95% air and 5% CO_2_. The day after transfection, 500 µl Opti-MEM medium were added to each well and finally, 48 hour after transfection, cell supernatants were transferred into clean microtubes. Protein concentration of each sample was measured by a non-specific ELISA. Next, for choosing the stably expressing clones, cells were grown in 0.7 mg/ml Hygromycin for 10-12 days and subcloned four times. In the end, stable clones were cultured in serum free medium at a large scale and their supernatants were collected for protein purification. Because of the presence of mouse Fc in the structure of HER3-Full ECD and its subdomains, we used HiTrap protein G HP column (GE Healthcare, Buckingham, UK) for purification of the expressed fusion proteins.

*Screening of protein expression by ELISA*

A non-specific sandwich ELISA using rabbit anti mouse Ig and HRP-conjugated rabbit anti mouse Ig (SinaBiotech Co., Tehran, Iran) was developed to detect the fusion proteins. Briefly, 50 µl of 5 µg/ml of rabbit anti mouse Ig, dissolved in PBS, were coated in a 96-well flat bottom microtiter plate (Maxisorp, Nunc, Roskilde, Denmark) and the plate was incubated at 37°C for 90 minutes. Then, the plate was washed with PBS-Tween 20 (0.05% Tween 20) for three times. Nonspecific binding sites were blocked for 90 minutes at 37°C with PBS-Tween 20 (0.05%) containing 3% skim milk (Merck, Darmstadt, Germany) and the plate was washed three times. Culture supernatants at different dilutions were added to wells and incubated in 37°C for 60 minutes. After three times washing, HRP-conjugated rabbit anti mouse Ig at suitable dilution was added and incubated at 37°C for another 60 minutes. After washing, tetramethylbenzidine (TMB) substrate (Pishtaz Teb, Karaj, Iran) was added in each well and the reaction was stopped by adding 1N HCL and then Optical Density (OD) was measured by an ELISA reader (BioTek, Winooski, VT, USA) at 450 nm.

*Rabbit immunization by recombinant proteins*

Female New Zealand white rabbits (Razi Vaccine and Serum Research Institute, Karaj, Iran) were immunized intramuscularly with 50 µg of HER3-Full ECD domain and subdomains dissolved in 500 µL of 1X PBS and emulsified in 500 µL of complete Frund’s adjuvant (Sigma, St Louis, MO, USA). The subsequent four booster injections with 25 μg of recombinant proteins in incomplete Freund’s adjuvant (Sigma) were given every two weeks after the primary immunization. Sera of the immunized rabbits were collected weekly. Titers of the polyclonal antibodies were assessed by ELISA. High-titred sera were purified by HiTrap protein G HP column. The animals were maintained based on Tehran University of Medical Sciences guideline for ethical use.

*Titration of immunized rabbit sera by ELISA*

Fifty microliters of 5 µg/ml of recombinant proteins were coated in 96-well plates and incubated for 90 minutes at 37°C. After three times washing with PBS-T, blocking was done for 90 minutes at 37°C. Then, serial dilutions of rabbit sera were added to the wells and incubated for 60 minutes at 37°C. After washing, HRP-conjugated sheep anti-rabbit IgG was added and incubated for 60 minutes at 37°C. After the final wash, the reaction was developed by using TMB substrate and after stopping the reaction, the absorbance was read at 450 nm.

*Purification of polyclonal antibodies*

Rabbit sera were purified in a two-step procedure. First, total rabbit IgG was purified by a HiTrap protein G HP column. Second, anti-HER3-Full ECD and subdomains were separated from anti-mouse Fc by using a Sepharose 4B column coupled with mouse IgG2a mAbs (prepared in our laboratory) (Hosseini-Ghatar et al., 2017a). 

*Confirmation of anti-mouse Fc adsorption from rabbit polyclonal antibodies by ELISA*

To confirm adsorption of anti-mouse Fc and isolation of HER3 specific antibodies, an ELISA test was carried out. 5 µg/ml of HER3 recombinant proteins and mouse Fc fragments were coated separately and incubated for 90 minutes at 37°C. After blocking with 3% skim milk, adsorbed polyclonal antibodies were added and incubated for 60 minutes at 37°C. HRP-conjugated sheep anti-rabbit Ig was added and incubated at 37°C for 60 minutes. The reaction was revealed by TMB, stopped by HCL 1N and read at 450 nm. 

*Purity determination and structural characterization of recombinant proteins by SDS-PAGE and immunoblotting assay*

Five µg of recombinant proteins were electrophoresed on 10% SDS–polyacrylamide gels under reducing and non-reducing conditions. Mouse IgG was used as a positive control. 

For immunoblotting, after protein electrophoresis on the SDS-PAGE gel, the proteins were transferred onto a nitrocellulose membrane (Schleicher & Schuell, Dassel, Germany). The membrane was blocked by PBS-Tween 20 (0.05%) containing 5% skim milk (Merck, Germany) for 60 minutes at RT. Mouse Fc-adsorbed polyclonal antibodies in a suitable concentration were added to their specific antigens and incubated overnight at 4°C. After three times washing with PBS-Tween 20 (0.05%) the membrane was incubated with HRP-conjugated sheep anti-rabbit at RT. In the end, positive protein bands were developed by ECL prime kit (Amersham Pharmacia Biotech, Chalfont, UK). 

*Cross-reactivity assessment of polyclonal antibodies with other HER family members*

An indirect ELISA was designed to determine cross-reactivity between anti-HER3 Full ECD and other HER family members (HER1, HER2 and HER4). Briefly, commercial recombinant HER1, HER2, HER3 and HER4 proteins (Speed BioSystems, Rockville, MD, USA) with 1 µg/ml concentration were coated in 96-well plates for 90 minutes at 37°C. HRP-anti-His tag was used for confirmation of the coating layer. After washing and blocking, anti-HER3 Full ECD and subdomains were added at different concentrations (10, 2.5, 0.625 µg/ml) to the plates and incubated at 37°C for 60 minutes. One hour incubation in the presence of HRP-conjugated sheep anti-rabbit Ig (SinaBiotech, Tehran, Iran) at 37°C was done and finally, the reaction was revealed by TMB and after stopping by HCl, the optical densities (OD) were measured by an ELISA reader at 450 nm.

*Analysis of reactivities of polyclonal antibodies with tumor cells by flowcytometry*

To prove that the produced polyclonal antibodies could bind to the native forms of HER3 receptors on tumor cell surfaces, we used flowcytometry assay on 3 breast cancer cell lines. BT-474, JIMT-1 and HCC-1954 cells (National Cell Bank of Iran) were trypsinized and washed twice with 1X PBS. The cells were then blocked with FACS 5% buffer (PBS 1X, 5% FBS) for 30 minutes at 4°C. Anti-HER3 full ECD antibody at 20 µg/ml concentration was added to 5×10^5^ cells and incubated at 4°C for 60 minutes. We considered anti-Full ECD HER2 (Hosseini-Ghatar et al., 2017b), PE-anti-human erbB3/HER-3 antibody (Clone 

1B4C3, Biolegend, USA) and non-immune rabbit IgG as positive and negative controls, respectively. Twice washing with PBS was done. After 45 minutes incubation with 5 µg/ml of FITC-conjugated sheep anti-rabbit Ig at 4°C, cells were washed again. In the end, 20×10^3^ cells were counted for each sample with a flowcytometer (Partec, Nuremberg, Germany) and the data was analyzed with FlowJo v10 software (Tree Star, Inc, Ashland, OR, USA). 

*Assessment of anti-tumor effects of polyclonal antibodies on breast cancer cell lines by XTT assay*

Anti-tumor effects of the produced anti-HER3 subdomain polyclonal antibodies were assessed on BT-474 and JIMT-1 cells. Five thousand cells/well were seeded in 96-well flat bottom plates in 100 µl RPMI-1640 medium containing 20% FBS for BT-474 and 10% for JIMT-1 and allowed to adhere overnight at 37°C. The cells were then treated with 100, 25 and 5 µg/ml of anti-HER3 Full ECD and 100 µg/ml of anti-subdomain antibodies and incubated an additional 72 hour at 37°C. 

Hundred µg/ml of HER2 Full ECD polyclonal antibody (Hosseini-Ghatar et al., 2017b) and 100 µg/ml of non-immune rabbit IgG were considered as positive and negative controls, respectively. After 72 hour incubation, we added 2, 3-bis (2 methoxy-4-nitro-5-sulfophenyl)-5-([phenylamino]-cabonyl)-2Htetrazolium (XTT) solution according to the instruction of the manufacturer (Roche Diagnostics, Mannheim, Germany). Four hours incubation in the presence of XTT was done and in the end, the microtiter plates were read by ELISA reader at 450 nm. Two different experiments were carried out in triplicates and tumor growth inhibition rates were estimated by the following formula:

Tumor growth inhibition (%) = (OD without antibody-OD with antibody) /OD without antibody × 100

*Statistical analysis*

In order to compare the groups, we used parametric one way ANOVA (Tukey test) and P values of less than 0.05 were taken as significant.

## Results

*Production of HER3-ECD domain and subdomain recombinant proteins*

**Figure 1:**
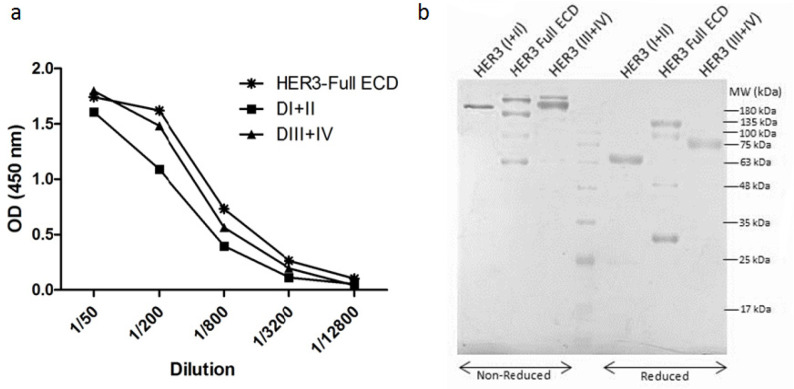
Characterization of Recombinant HER3 ECD Subdomains. a) Detection of recombinant proteins in culture supernatant of stable transfected CHO-K1 cells; b) Electrophoresis pattern of purified recombinant proteins. Purified proteins were resolved on 10% SDS-PAGE gel under reducing and non-reducing conditions and then stained with Coomassie blue. SM, size marker (kilodalton).

After transfection of CHO-K1 host cells with HER3-Full ECD and its subdomain constructs, HER3-Full ECD, DI+II and DIII+IV fusion proteins were successfully produced in these cells (Figure 1a). Considering the presence of mouse Fc γ2a fragment in the structure of the recombinant proteins, protein expression could be detected in cell supernatants using mouse Ig specific polyclonal antibodies by ELISA.

*Characterization of the purified recombinant proteins by SDS-PAGE*

After purification of recombinant proteins from serum-free culture supernatants by protein G column, the purity and structure of the purified proteins were assessed by SDS-PAGE. Coomassie blue staining of SDS-PAGE gel showed high purity of the recombinant proteins and because of glycosylation in the mammalian host cells and dimerization by IgG2a hinge, all purified proteins had higher molecular weights compared to their expected amplicon size (Figure 1b). Different HER3-Full ECD size bands observed in reduced and non-reduced conditions are probably related to monomer, polymer or degraded forms. According to Table 1, the amplicon size of DI+II and DIII+IV is almost similar with a molecular weight of approximately 36 Kd. Since the hinge sequence of mouse Fc region is present in the construct leading to dimerization of the expressed proteins, the final molecular weight of the assembled fusion proteins would be around 206, 134 and 137 Kd for ECD-full HER3, DI+II and DIII+DIV, respectively, in non reducing form. Moreover, the molecular weight of DIII+IV was found to be higher than DI+II. According to the glycosylation site prediction (NetNGlyc 1.0 Server, http://www.cbs.dtu.dk/services/NetNGlyc), there are 4 times higher possible glycosylation positions in DIII+IV compared to DI+II which could justify its higher molecular weight.

*Production and purification of polyclonal antibodies*

**Figure 2:**
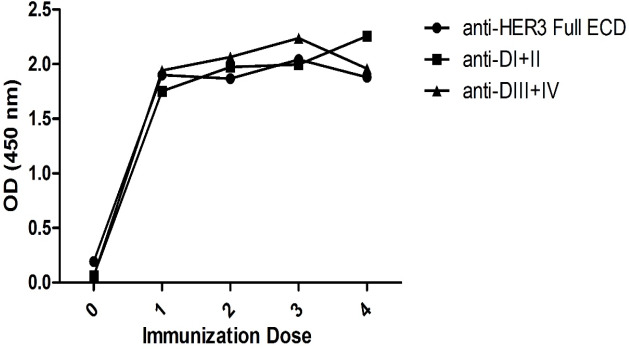
Titration of Serum from Rabbits Immunized with Recombinant HER3-ECD Proteins. After administration of each immunization dose, serum samples from the immunized rabbits were collected and tested at 1/800 dilution against the corresponding immunogens

The purified proteins were injected to rabbits and serum samples were collected after each immunization. Reactivities of the antibodies were tested against the corresponding antigens at 1/800 dilution after each immunization. The results indicated that the high titers of antibodies against HER3-Full ECD, DI+II and DIII+IV subdomains reached plateau after administration of the first immunization dose and slightly increased after subsequent booster doses (Figure 2). 

**Figure 3:**
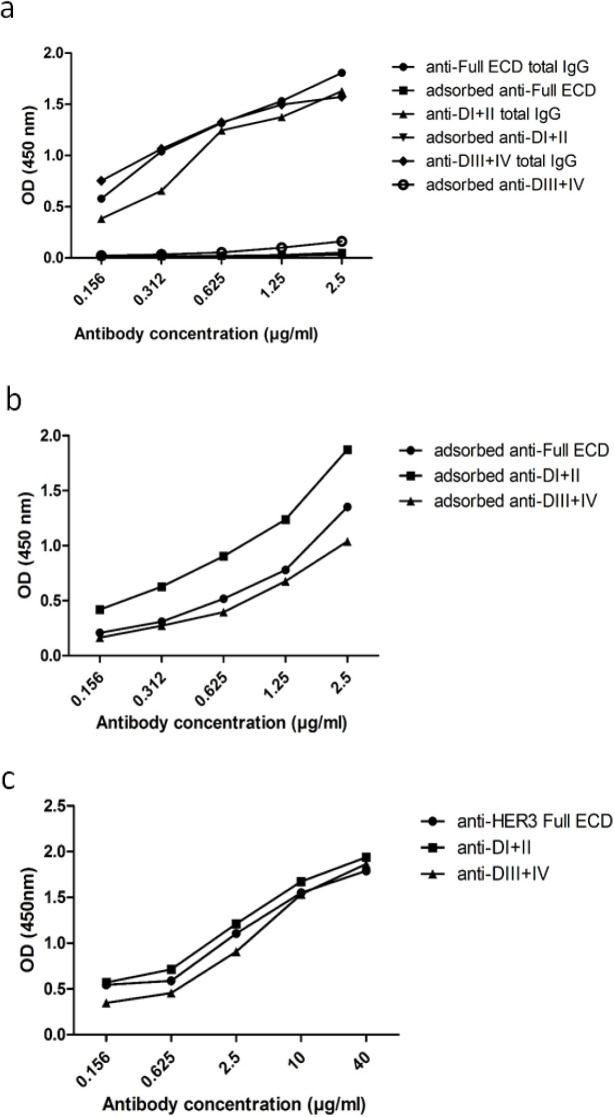
Reactivity of Purified Anti-HER3 ECD Proteins before and after Adsorption. a) Polyclonal antibodies were adsorbed with IgG2a and the reactivity of the antibodies was assessed on an IgG2a mAb before and after adsorption; b) Adsorbed antibodies were titrated on the corresponding immunizing recombinant proteins, or c) HER3 full ECD

After last immunization, rabbit sera were collected and purified in two steps. In the first step, total rabbit IgG was purified by protein G column and then specific rabbit anti-mouse Fc antibodies were removed by a Sepharose 4B column coupled with mouse mAbs. The cross reactivities of these adsorbed anti-HER3 Full ECD and subdomain antibodies were assessed by ELISA. The results showed that total IgG from immunized rabbits reacted with mouse IgG Fc fragments, but after anti-mouse Fc removal, adsorbed polyclonal antibodies could only react with their specific antigens, but not with mouse Fc fragments [Figure 3(a and b)]. Adsorbed polyclonal antibodies against DI+II and DIII+IV could recognize HER3 Full ECD protein in the same manner as anti-HER3-ECD antibody [Figure 3(c)].

*Immunoblotting of purified HER3 ECD domain and subdomain specific polyclonal antibodies*

**Figure 4:**
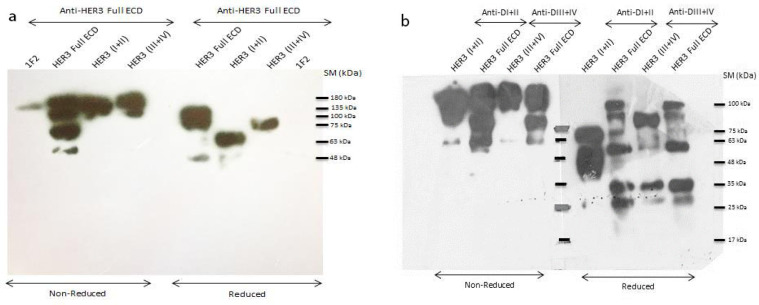
Immunoblotting of HER3 ECD Domain and Sub-Domains Specific Polyclonal Antibodies. After resolving the recombinant proteins on 10% SDS-PAGE under reducing and non-reducing conditions, the bands were transferred to a nitrocellulose membrane and revealed with anti-HER3 Full ECD (a) or anti-DI+II and -DIII+IV (b) polyclonal antibodies. 1F2 is a mouse IgG2a monoclonal antibody which is employed as a control. SM: size marker (kilodalton)

Five µg of each recombinant protein and a mouse IgG2a monoclonal antibody (1F2) (Tahmasebi et al., 2014) were run on SDS-PAGE gel under reduced and non-reduced conditions and then trasferred to nitrocellulose membrane as described in Materials and Methods. The results showed that the adsorbed anti-HER3 full ECD antibody could recognize both HER3 full ECD and the two other subdomains. Since anti-HER3 full ECD was adsorbed, negligible reactivity with mouse IgG2a was observed. The immunoblotting profile is very similar to the SDS-PAGE results with the exception of the ~30Kd band observed in the reduced HER3 full ECD lane which is absent in the corresponding WB results. This band most likely represents the Fc fragment which could not be picked up by the Fc IgG adsorbed anti-HER3 full ECD antibody (figure 4a). We also investigated the reactivities of anti-DI+II and -DIII+IV with their specific antigens and HER3 full ECD protein. The results indicated that the adsorbed anti-DI+II and anti-DIII+IV polyclonal antibodies could recognize their coresponding subdomains as well as HER3 full ECD (Figure 4b). The WB profile obtained with these anti-subdomain antibodies is somehow different from the SDS-PAGE profile. This could be due to utilization of two different preparations of purified fusion proteins for these experiments. The preparations employed in figure 1b is the same as that used in figure 4a, but different from the preparations employed in figure 4b. The latter preparations imply that the purified proteins were slightly degraded after purification, leaving more bands in the WB results. Similar to the SDS-PAGE results, purified DIII+DIV had a higher molecular weight than DI+II protein. 

*Cross-reactivity with other HER family members*

**Figure 5.**
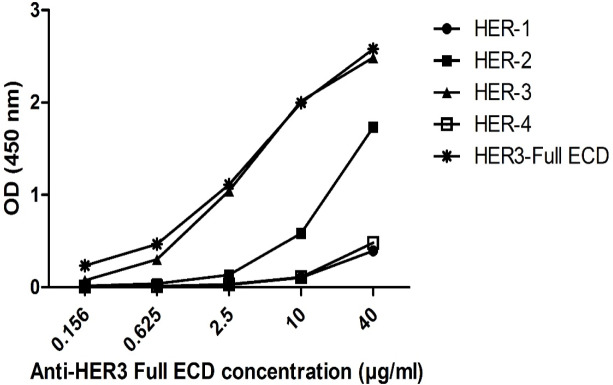
Cross Reactivity of Anti-HER3 Full ECD with HER Family Members. Adsorbed anti-HER3 full ECD antibody was tested against 2.5 µg/ml of four commercial recombinant HER family members as well as HER3 full ECD prepared in this study

Because of homology between HER family members, we carried out an ELISA to determine if anti-HER3 Full ECD could react with other HER family members. So we titrated polyclonal anti-HER3 Full ECD on commercial recombinant HER1, HER2, HER3, HER4 proteins along with our purified HER3 Full ECD fusion protein. The results indicated that the adsorbed anti-HER3 full ECD could recognize our purified HER3-ECD similar to the commercial HER3-ECD (Figure 5). No or negligible reactivity was observed with HER1 and HER4 proteins, but weak to moderate reactivity was noticed to HER2 molecule. 

*Binding of HER3-ECD specific polyclonal antibody to three different breast cancer cell lines*

**Figure 6:**
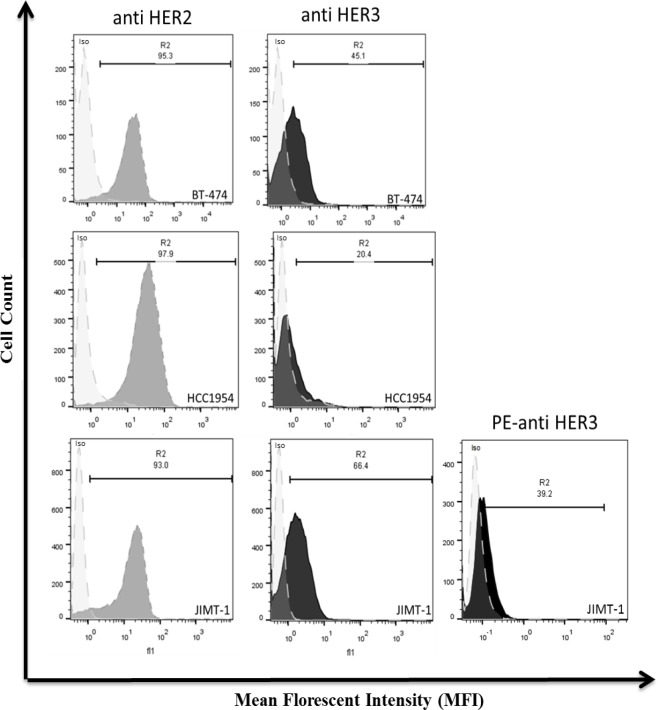
Flowcytometry Profile of Anti-HER3 Full ECD Antibody in Breast Cancer Cell Lines. BT-474, JIMT-1 and HCC-1954 cells were stained with adsorbed anti-HER3 Full ECD antibody as primary and FITC-labeled sheep anti-rabbit Ig as secondary antibody. Normal rabbit IgG at the same concentration was used as a negative control. The commercial PE-anti HER3 was also used as a positive control

We used flowcytometry assay to determine the binding of anti-HER3 full ECD to HER3 receptors on the surface of BT-474, JIMT-1 and HCC-1954 breast cancer cells lines. Because of high level of expression of HER2 on these cell lines, we used anti-HER2 Full ECD polyclonal antibody (Hosseini-Ghatar et al., 2017b) as a positive and non-immune rabbit IgG as a negative control. Although the flowcytometry results showed that all breast cancer cell lines were stained with anti-HER3 polyclonal antibody, the percentage of positive cells and MFI were less than those stained with anti-HER2 antibody (Figure 6). JIMT-1 and BT-474 had higher expression of HER3 on their surfaces and were then selected for the next experiments. 

*Tumor growth inhibition by anti-HER3 subdomain antibodies*

**Figure 7:**
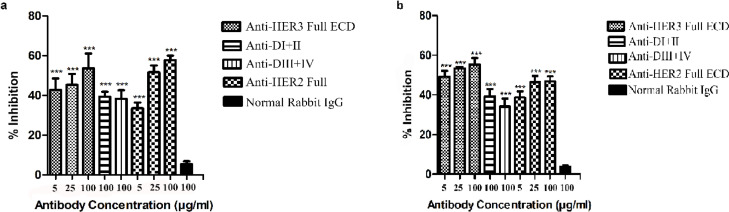
Growth Inhibition of HER3 Positive Breast Cancer Cells by Anti-HER3 Polyclonal Antibody. BT-474 (a) and JIMT-1 (b) breast cancer cells were treated with different concentrations (5, 25 and 100 μg/ml) of anti-HER3 Full ECD, 100 μg/ml of anti-subdomain antibodies, 100 µg/ml of irrelevant non-immune rabbit IgG (negative control) and 100 μg/ml of anti-HER2 Full ECD (positive control). The results represent mean and standard deviation of percent inhibition obtained by XTT assay for three independent experiments. (***) shows significant growth inhibition (p<0.001) of different treatments compared with negative control

Anti-tumor effects of adsorbed polyclonal antibodies were assessed by XTT assay on growth of breast cancer cell lines. The results demonstrated that the anti-HER3 subdomain polyclonal antibodies significantly inhibited the growth of BT-474 and JIMT-1 cell lines (Figure 7). 

**Table 1:** List of Primers Used for Amplification of HER3-ECD and Its Subdomains

Domain	Primer	Sequence	Amplicon size (bp)
Full HER3-ECD	HER3-Full-F	ATATTCCCGGGTAAACTCTCAGGCAGTGTGTCC	1929 bp
	HER3-Full-R	ATAATTCGAATGTCAGATGGGTTTTGCC	
DI+II	HER3-Full-F	ATATTCCCGGGTAAACTCTCAGGCAGTGTGTCC	897 bp
	HER3-DI+II-R	ATAATTCGAAAGGCTCACACATCTTG	
DIII+IV	HER3-DIII+IV-F	ATATTCCCGGGTAGACAAGATGGAAGTAG	963 bp
	HER3-Full-R	ATAATTCGAATGTCAGATGGGTTTTGCC	

## Discussion

EGF receptor family is a major cell growth and differentiation regulator and any disturbance in this family may lead to a variety of cancers. Contrary to HER1 and HER2, which have been widely studied, HER3 was neglected for a long period of time due to possession of inactive kinase domain. Recent studies have demonstrated the role of HER3 in signalling and evaluated its importance in several clinical studies (Sergina and Moasser, 2007; Campbell et al., 2010; Kol et al., 2014). The presence of six phosphotyrosine residues in HER3 carboxyl tail has pointed it as an intrinsic activator of PI3K-Akt pathway. PI3K-Akt signalling is associated to tumorgenesis and lack of control of this pathway has been reported in many types of cancer (Sithanandam and Anderson, 2008; Gala and Chandarlapaty, 2014). Although many therapeutic agents have been produced against tyrosine kinase receptors, in many cases, resistance to treatment has been reported. HER3, with its compensatory signalling potential in combination with TKI inhibitors, plays a critical role in PI3K/Akt activation and following the treatment failure (Sergina et al., 2007; Baselga and Swain, 2009). In addition, HER3 is a preferred partner for HER2 which makes it the most active heterodimer in this family. Therefore, development of targeted therapeutics against HER3 is a promising tool for treatment of many HER3 overexpressing cancers (Ma et al., 2014). A large number of monoclonal antibodies (mAbs) have been generated against HER3 but non has so far received therapeutic approval (Jiang et al., 2012; Malm et al., 2016). Taking into consideration the fact that some epitopes located in different ECD subdomains of HER3 are biologically important because of their influence on ligand binding and receptor dimerization, therefore, targeting these epitopes is crucial for targeted therapy. Each mAb recognizes and attaches randomly to a specific epitope within a given subdomain and thereby affects the downstream signalling function of HER3. Some of these mAbs display inhibitory function and are considered as a potential therapeutic arsenal. Interestingly, the combination of some selected HER2 specific mAbs has shown synergistic inhibitory effects on tumor cells and are proposed for combination therapy. Approval of Pertuzumab by FDA as a combination therapy together with Trastuzumab in HER2 overexpressing metastatic breast cancer patients is an example of these mAbs (Scheuer et al., 2009; D’Souza et al., 2014; Yonesaka et al., 2016). We have recently produced a novel humanized mAb (Hersintuzumab) which displays superior tumor inhibitory activity in combination with Trastuzumab as compared to the combination of Pertuzumab and Trastuzumab (Amiri et al., 2018).The same principle may be applicable for HER3 specific mAbs. 

Based on these observations we decided to express HER3 ECD subdomains as paired I+II and III+IV subdomains in a eukaryotic system and produce polyclonal antibodies trying to find out whether these antibodies can mimic the combination strategy and display differential inhibitory activity on HER3 expressing tumor cell lines. We did not construct single subdomains of HER3 ECD, because our previous experience with HER2 had shown that these isolated subdomains lacked some tertiary and quaternary structures leading to induction of polyclonal antibodies which either failed or weakly reacted with the native HER2 molecule (Hosseini-Ghatar et al., 2017a). We also encountered a similar problem with recombinant HER2 subdomain proteins expressed in prokaryotic system (Sadri-Ardalani et al., 2017). 

The paired subdomains of HER3 were fused to the mouse Fc γ2a gene to yield a fusion protein with higher immunogenicity, solubility and easy to purify using protein A or protein G column. Indeed, the presence of this tag enabled us to largely avoid aggregation of eluted proteins and collection of highly purified preparations with >95% purity, as judged by SDS-PAGE (Figure 1). These purified proteins were employed to produce polyclonal antibodies in rabbits. Serum titration of the immunized rabbits showed efficient immunization with both paired HER3 sub-domains and full HER3 ECD (Figure 2). The antibody preparations were adsorbed with IgG2a mAbs to remove anti-mouse Fc molecules. Adsorbed antibodies strongly reacted with the immunogens as well as the full HER3 ECD molecule, but failed to bind to mouse IgG2a (Figure 3). These results clearly indicated that our strategy worked fairly well and the purified polyclonal antibodies could be used to target HER3 positive tumor cells. The cross-reactivity experiment with other HER family members indicate lack of reactivity with HER1 and HER4 and moderate to weak reactivity with HER2 (Figure 5). Of the three HER2 positive tumor cell lines tested with anti-HER3 full ECD antibody, two displayed moderate positive patterns (Figure 6), suggesting specific reactivity of the antibody at the specified concentration with only HER3, but not HER2 molecules. These two positive cell lines (BT-474 and JIMT-1) were selected for XTT assay to assess tumor inhibitory potential of the polyclonal antibodies. Interestingly, both HER3 full ECD and to a lesser extent HER3 paired subdomains induced 40-60% growth inhibition which was dose dependent (Figure 7). There seems to be no difference between I+II and III+IV subdomains specific polyclonal antibodies with regards to their inhibitory activity. Thus, both proteins are suitable for either passive or active targeted therapy of HER3 positive cancer types, particularly breast cancer. This is very promising because tumor cells from 50-70% of these patients express HER3 which is higher than the frequency of HER2 overexpressing tumors in breast cancer patients (Ocana et al., 2012).
